# The Doctor at Burlington House

**Published:** 1913-05-24

**Authors:** 


### THE DOCTOR AT BURLINGTON HOUSE.

### Hospital Interest at the Academy.

From the point of view of the anatomist, the Academy Exhibition is always interesting, though hardly as much can be said for its medical and institutional interest. Yet it is a noteworthy fact that every year the Academy contains more pictures that may be supposed to attract the attention of the medical man who visits it than did the Exhibition of the preceding year. We may take this as a tribute to the growing interest felt by the general public in things medical, an interest that is reflected in the daily press and in contemporary plays. There was a time when the Academy hardly admitted a medical portrait, and when morbid "medical stuff," as one critic styled it, was banned. We have changed all that since Fildes's "The Doctor" was the show picture of its year, and to-day it is by no means uncommon to find an Academy exhibition that teems with pictures and portraits which have a more particular interest to institutional men.

This year's display is, unfortunately, somewhat of an exception to this generalisation. There is not one picture that can be said to have as great a professional interest as "The Doctor." We have to look at the portraits and a few of the subject-pictures to find matters of interest. Taking the portraits first, undoubtedly the best of those which have a more peculiarly medical interest is Mr. R. Eve's bust length of Dr. Norman Moore. Those who know our foremost authority on medical history will easily recognise the characteristic attitude and expression; the portrait is not, perhaps, worthy to rank among the great portraits in the exhibition—such as Herkomer's portrait of Lord Morley—but it is a pleasing colour study, painted with a Holbein-like simplicity and directness that must appeal. In the same room hangs Sir Hubert von Herkomer's portrait of Sir Berkeley Moynihan, the background of it inscribed "To my friend and benefactor." It is admirably painted, like most of Herkomer's canvases, but sombre in colour with little relief, while there is as little in the face or figure to call for comment. In Room IV. Sir Hubert has another portrait, this time of his physician, and inscribed "From his grateful patient"; it is equally well painted, but the visitor, whether lay or medical, almost at once allows his glance to stray to Mr. Carlo's "Scout" subject, which hangs above the practitioner's portrait and is entitled "Good Service Work in a London Slum." In Room II. is what appears to us the finest picture of medical interest in the Academy this year. It is Mr. Sargent's brilliant little sketch of the loggia of the general hospital at Granada, showing the patients clustered round the pillars. There is admirable contrast in the work, and the patient on the low stretcher in the foreground is painted with a detail that makes it one of the most realistic, and at the same time most interesting, hospital studies we have seen. Mr. Sargent's picture shows what artists may discover in our hospitals, and how splendidly they can portray it, provided they have the genius and the skill to rival his brilliant technique and mastery of light and shade. In Room III. is Mr. Ouless's portrait of Dr. Hayes Newington, treasurer of the Medico-Psychological Association; it is well painted, with strong contrasts, but lacks the simplicity and dignity of Mr. Eve's representation of Dr. Moore.

In Room VII. hangs Mr. Nowell's portrait of Sir Thomas Chavasse; and in the next room is a fine study, by Mr. Peacock, of Dr. Kinsey-Taylor's little daughter. In the Sculpture Galleries there is a good deal of excellent work shown this year. No. 1,795, entitled "Famine Relief," and destined to form part of the Curzon Memorial at Calcutta, is by Mr. Thornycroft, and is a very forcible and realistic work of art. No. 1,867 is the bronze memorial to the late Dr. Huggard, of Davos, one of the founders and for twenty-six years superintendent of the Queen Alexandra Sanatorium at Davos. Mr. F. Arnold White, the sculptor, has admirably succeeded in his endeavour to give the two-figure group which surmounts the tablet that appearance of calm restfulness combined with strength which appeals to the spectator when viewing such memorials. Below is Sir Thomas Brock's portrait bust of Lord Lister, destined for the Royal College of Surgeons; it is a skilful and artistic representation of the great surgeon as some of his younger contemporaries remember him when he was at the height of his fame and skill—the kind mouth, the firm chin, and the broad forehead shading the placid eyes—and is a worthy memorial to add to the gallery that the College already possesses in its fine series of busts of our great surgeons. Miss Knoblauch's little statuette of Florence Nightingale (No. 1,941) is a very delicate piece of work, and hardly gives the spectator the impression of calm strength conveyed by Mr. Walker's statue (which has not yet been exhibited in public), but its refined beauty and grace will please all those who admire the "Lady with the Lamp." Close by is Mr. Forsyth's excellent bust of Mr. William A. Pite, the architect of King's College Hospital.

In the architectural room are many drawings of great interest. Sir T. G. Jackson, Bart., shows an elevation of the new entrance hall of the Physiological Laboratory at Cambridge, and Mr. Stratton has an interesting sketch of the new King's College Schools' science block, the ground plan of which is simple and admirably designed. Messrs. Young and Hall show plans of the new reconstruction of the Chelsea Hospital for Women, and Mr. Charles Smith has a plan of the Bradfield College Science Laboratories. The most instructive plans, however, are those of new houses which clearly show how simplicity and comfort, with a due regard for hygienic considerations, have become guiding lines in domestic architecture. This room will well repay a visit and careful study by those interested in architectural questions.

